# The Gulf of Aden Intermediate Water Intrusion Regulates the Southern Red Sea Summer Phytoplankton Blooms

**DOI:** 10.1371/journal.pone.0168440

**Published:** 2016-12-22

**Authors:** Denis Dreano, Dionysios E. Raitsos, John Gittings, George Krokos, Ibrahim Hoteit

**Affiliations:** 1 Computer, Electrical and Mathematical Sciences & Engineering Division, King Abdullah University of Science and Technology, Thuwal, Saudi Arabia; 2 Remote Sensing Group, Plymouth Marine Laboratory, Plymouth, United Kingdom; 3 National Centre for Earth Observation, Plymouth Marine Laboratory, Plymouth, United Kingdom; 4 Biological and Environmental Science & Engineering Division, King Abdullah University of Science and Technology, Thuwal, Saudi Arabia; 5 Physical Science and Engineering Division, King Abdullah University of Science and Technology, Thuwal, Saudi Arabia; Fisheries and Oceans Canada, CANADA

## Abstract

Knowledge on large-scale biological processes in the southern Red Sea is relatively limited, primarily due to the scarce *in situ*, and satellite-derived chlorophyll-a (Chl-a) datasets. During summer, adverse atmospheric conditions in the southern Red Sea (haze and clouds) have long severely limited the retrieval of satellite ocean colour observations. Recently, a new merged ocean colour product developed by the European Space Agency (ESA)—the Ocean Color Climate Change Initiative (OC-CCI)—has substantially improved the southern Red Sea coverage of Chl-a, allowing the discovery of unexpected intense summer blooms. Here we provide the first detailed description of their spatiotemporal distribution and report the mechanisms regulating them. During summer, the monsoon-driven wind reversal modifies the circulation dynamics at the Bab-el-Mandeb strait, leading to a subsurface influx of colder, fresher, nutrient-rich water from the Indian Ocean. Using satellite observations, model simulation outputs, and *in situ* datasets, we track the pathway of this intrusion into the extensive shallow areas and coral reef complexes along the basin’s shores. We also provide statistical evidence that the subsurface intrusion plays a key role in the development of the southern Red Sea phytoplankton blooms.

## 1. Introduction

The Red Sea is an elongated (~2250 km) oceanic basin situated between Asia and Africa. The southern end of the basin is connected with the open Indian Ocean via the narrow strait of Bab-el-Mandeb. As a result of its geographical position at subtropical latitudes, isolation, lack of riverine input and low precipitation rates (2 cm / year), the Red Sea is one of the warmest and most saline marine environments in the world [1]. Despite these extreme conditions, the Red Sea accommodates one of the world's largest coral reef complexes, while supporting an ecosystem characterised by high endemism and biodiversity [2,3].

There is a marked physicochemical gradient along the north-south axis of the Red Sea [4,5], which has a profound impact on its biology [6]. The southern Red Sea coastal areas are characterised by shallow banks and turbid waters, and differ biologically from the rest of the basin, in that they contain extended mangrove habitats, less developed coral reefs and an increased presence of seagrasses and macroalgae [4,7]. Due to the proximity of the Bab-el-Mandeb strait connecting it to the Indian Ocean, the southern Red Sea waters are less saline and more nutrient-rich [4,8,9]. This area is considered the most productive part of the Red Sea, where intense phytoplankton blooms have been observed [6,10,11].

Phytoplankton form the base of the marine food chain and therefore play a crucial role in marine ecosystems, including coral reefs [10]. The functioning of coral reef ecosystems relies on these microscopic marine algae, which provide a source of food for many coral reef-associated organisms, including zooplankton, sponges, bivalves, and free-swimming larvae. Thus, any changes in phytoplankton bloom timing or abundance may lead to trophic mismatch and alter the functioning of marine ecosystems [12,13]. For example, a significant positive relationship between satellite-derived Chl-a (an index of phytoplankton biomass) and the recruitment success of coral reef fish larvae has been reported in French Polynesia [14].

Previous studies using remotely-sensed measurements of Chl-a have suggested that the Red Sea is a winter/spring blooming environment [6,15,16], although indications of summer blooms have also been reported in the southern Red Sea [6]. An *in situ* study off the coast of Jeddah in the central Red Sea also suggested that a summer phytoplankton bloom of cyanobacteria (*Trichodesmium* spp.) occurs between June and August [17]. Unfortunately, adverse atmospheric conditions (haze and clouds) during this period have severely limited the retrieval of satellite Chl-a data in this area and prevented further investigation [6,11]. Recently, Chl-a data coverage in the southern Red Sea has been substantially improved by a new ocean colour data product that combines observations from three different sensors: the European Space Agency (ESA) developed Ocean Colour Climate Change Initiative (OC-CCI) [11].

A recent study using OC-CCI revealed that most of the reef-bound coastal waters of the southern Red Sea experience high Chl-a concentrations during summer (at the onset of the South Asian summer monsoon) [11]. Despite their importance, these summer blooms have not been studied thoroughly and the mechanisms regulating them remain unknown. Using the enhanced data coverage of the OC-CCI product, we assess the spatiotemporal distribution of the southern Red Sea summer Chl-a concentrations and their links with the regional environmental conditions. To understand the physical processes triggering these blooms, we explore the hypothesis of Churchill et al. [18] who proposed that a subsurface intrusion of nutrient-rich water from the Gulf of Aden into the Red Sea during summer could play an important biological role for the southern Red Sea ecosystem. Using remotely-sensed, modelled and in situ datasets, as well as climate indices, we investigate the link between the southern Red Sea summer phytoplankton blooms and the subsurface intrusion of the Gulf of Aden Intermediate water (GAIW).

## 2. Methods

### 2.1 Datasets

#### 2.1.1 Satellite Remote Sensing datasets

**Fig 1:**
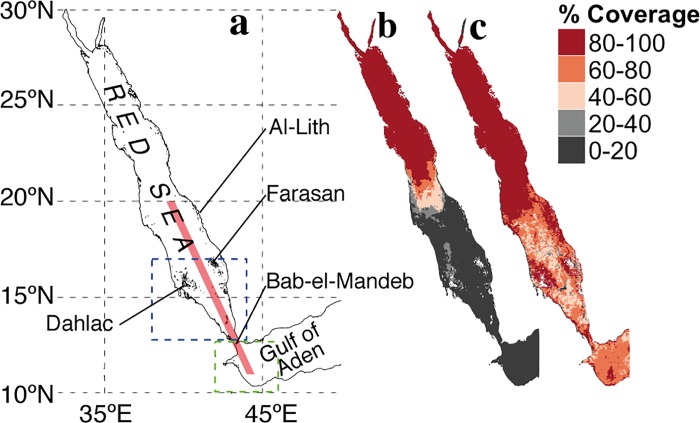
Schematic representation of the Red Sea, and percentage data coverage of satellite Chl-a data. ***a)*** Geographical locations in the Red Sea. Delimitations of the southern Red Sea (south of 17°N, blue dashed rectangle), and the western Gulf of Aden (west of 46°E, green dashed rectangle). The elongated pink rectangle represents the area over which the model outputs were averaged to obtain the vertical profiles (Fig 2). ***b)*** MODIS percentage data coverage in monthly composites for July (2002–2012). ***c)*** Similar to ***b***, but for OC-CCI merged sensor product data.

Monthly and 8-day Level 3 remotely-sensed surface Chl-a measurements from the OC-CCI dataset were acquired at a 4 km resolution. We extracted the data over the Red Sea and the Gulf of Aden (Fig 1A) for the period 2000 to 2012 from www.esa-oceancolour-cci.org. This merged product—produced and validated by the ESA—is the most complete and consistent time-series of multi-sensor global Chl-a data. It combines datasets from the Moderate Resolution Imaging Spectroradiometer (MODIS) Aqua, the Sea-Viewing Wide Field-of-View Sensor (SeaWiFS), and the Medium Resolution Imaging Spectrometer (MERIS). It has been corrected for bias and allows the retrieval of high-resolution Chl-a data with substantially improved coverage in the southern Red Sea region [11,19]. To display the improved data coverage between single sensors and the OC-CCI dataset, we compared OC-CCI data with MODIS Level 3 Chl-a data. Monthly composites for the period 2000 to 2012 were downloaded from the NASA OceanColor archive website (oceandata.sci.gsfc.nasa.gov). Regarding the MODIS dataset, the presence of clouds and haze during summer has resulted in very few observations over the southern Red Sea (Fig 1B). Indeed, the MODIS sensor appears to have severe issues in retrieving measurements of Chl-a during July (2002 to 2012; Fig 1B). One advantage of the OC-CCI dataset is the improved spatial and seasonal coverage in the Gulf of Aden and Red Sea (Fig 1C), which permits the investigation of summer Chl-a variability in the southern Red Sea. Overall, the average percentage data coverage for July (2002–2012) in the southern Red Sea (blue-box area average, Fig 1A) increased from ~5% for MODIS to ~64% for CCI.

Sea surface temperature (SST, °C) is an important parameter for studying phytoplankton dynamics. In tropical seas, like the Red Sea, colder SST can be indicative of upwelled nutrient-rich waters [6]. Here, we used a Level 4 (gap-free), blended dataset that merges day and night SST from the Advanced Very High Resolution Radiometer (AVHRR) [20]. The data have been corrected with *in situ* observations (acquired from buoys and ships), and mapped on a 0.25°x0.25° resolution grid using optimal interpolation [21]. Monthly and daily aggregates for the period 2000 to 2012 were obtained from podaac.jpl.nasa.gov. The daily SST aggregates were then averaged over the same 8-day periods as those of the remotely-sensed Chl-a dataset.

#### 2.1.2 Potential bias in remotely-sensed ocean colour observations

Large areas in the southern Red Sea are optically complex, in that they may contain suspended sediments, yellow substances, particulate matter, and/or coloured dissolved organic matter that do not covary in a predictable manner with Chl-a, thus limiting the application of remotely-sensed data [22]. In addition, the southern Red Sea has extended areas with shallow bathymetry, where the Chl-a signal may be obscured by the effect of seafloor reflectance [22]. A combination of these factors can result in an overestimation of Chl-a in the southern Red Sea [6,11,19]. However, validation studies using a suite of univariate statistical tests have shown a reasonable agreement between satellite-derived and independent in situ Chl-a data in the Red Sea with a relative root mean square error of 46% compared to 77% for the global ocean [23,24]. In addition, further recent validation studies [11,19] have specifically evaluated the performance of ocean colour data in shallow, reef-bound coastal waters in the Red Sea. Their performance over these areas (Pearson correlation coefficient r = 0.82) was found to be comparable to results observed in deeper ocean waters (r = 0.84), indicating that the use of ocean colour data is appropriate to study the relative increase of biological activity in the broader southern Red Sea during summer. The scope of the current study is to assess the general phytoplankton spatiotemporal variability at the southern part of the basin, regardless of absolute concentrations.

#### 2.1.3 Climate Indices

It is well documented that the seasonal monsoon winds regulate the winter Red Sea phytoplankton by enhancing the influx of nutrient-rich Gulf of Aden surface water [6,15,16]. In this study, we use the Indian monsoon index (IMI) (apdrc.soest.hawaii.edu/projects/monsoon/seasonal-monidx.html)—which has been linked to the air-sea heat exchange in the Red Sea [25]—to investigate whether the strength and variability of the South Asian monsoon regulates Chl-a concentrations in the southern Red Sea during summer. The index is defined as the difference in zonal winds (at 850 hPa) between two regions over the Indian Ocean and Northern India (5°-15°N, 40°-80°E and 20°-30°N, 70°-90°E) [26]. Daily values were acquired and aggregated to construct monthly and 8-day (corresponding to those of OC-CCI and SST) averaged time series for the period 2000 to 2012 (apdrc.soest.hawaii.edu/projects/monsoon/realtime-monidx.html). We also considered the use of the multivariate El-Niño southern oscillation index (MEI, www.esrl.noaa.gov/psd/enso/mei/). Despite its usefulness in describing the relationship between warmer global climate phases and higher Chl-a concentrations during winter [15], we found that the MEI was not significantly related to southern Red Sea Chl-a during summer.

#### 2.1.4 Research cruise datasets

**Fig 2:**
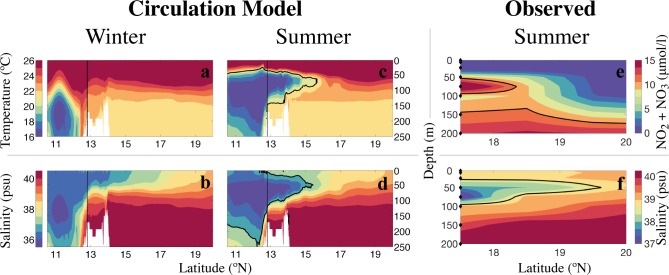
Vertical profiles of model outputs and *in situ* ship-borne observations, depicting the summer influx of colder, fresher, and nutrient-rich intermediate water masses (GAIW) into the southern Red Sea. ***a)*** and ***b)*** Profiles of temperature and salinity climatologies during winter (Jan-Feb), obtained from the MITgcm circulation model, and averaged over the transect indicated in Fig 1A. ***c)*** and ***d)*** Similar profiles for the summer period (Jul-Aug). ***e)*** and ***f)*** *In situ* measurements of nutrient concentrations and salinity during the summer 2011, aggregated in cells of one-degree width and centered at approximate depths of 5, 10, 25, 50, 75, 100 and 200 m (marked by black diamonds on the y-axis).

We used available in situ measurements of salinity and nutrients (NO_3_+NO_2_) acquired from Leg 1 of the King Abdullah University of Science and Technology (KAUST) 2011 Red Sea Expedition (R/V Aegaeo, September 15–October 10). Samples were taken at 206 stations along 20 transects across the Red Sea basin (between 17°N and 28°N). Further details regarding the data collection and processing are provided by Churchill et al. [18]. To show the intrusion of nutrient-rich water from the Gulf of Aden, we calculated the averages of salinity and nutrient in situ measurements in cells of one-degree latitude width and centered at approximated depths of 5, 10, 25, 50, 75, 100, 150, and 200 m. This division of the depth–latitude profile allowed us to obtain meaningful profiles of nutrients and salinity without data gaps (see Fig 2E and 2F).

#### 2.1.5 Modeled datasets

Outputs from a high resolution (~1.8 km) general circulation ocean model were used to compute the climatological profiles of salinity and temperature. The model was specifically configured to study of the general circulation of the Red Sea and presented in Yao et al. [27,28]. The hydrodynamics were simulated using the MIT General Circulation Model (MITgcm) over a 50-year period between 1952 and 2001. The model domain covers the entire Red Sea (including the Gulf of Suez and the Gulf of Aqaba) and part of the Gulf of Aden. The bathymetry was derived from the ETOPO2 dataset. The model was forced with 6 hourly atmospheric data from the NCEP/NCAR 40 year reanalysis 1 project (NCEP) [29], while open boundary conditions in the Gulf of Aden were specified by the reanalysis of the Estimation of the Circulation and Climate of the Ocean (ECCO) consortium [30]. The simulation was initialized from a state of rest using annual mean temperature and salinity from the World Ocean Atlas 2013 [31,32]. The model employed a 10-year spin up period, using the atmospheric conditions of 1952. The results and validation of these simulations were presented in Yao et al. [27,28]. Of specific interest for the current study, the seasonal exchange flow patterns and the volume transports at the strait of Bab-el-Mandeb, as well as the structure of the intruding flow, were found to be robust features of the 50 year run and consistent with available observations [27]. The model outputs have also been used to study the seasonal [27,28] and eddy [33] variability in the Red Sea.

Atmospheric forcing plays a key role in the Red Sea: they govern the air-sea heat fluxes [34] and the deep ventilation of this area, thus significantly influencing phytoplankton biomass [34,35]. Previous research has also suggested that the monsoon wind regime is an important factor governing phytoplankton biomass in the Red Sea [6,15,16]. Monsoon winds have been shown to be critical drivers of phytoplankton seasonal variability in other subtropical areas such as Sanya Bay (South China Sea), where the occurrence of summer and winter blooms correspond to monsoon winds [36]. Wind fields from a high resolution, downscaled assimilated product [37,38] were used to analyse the atmospheric variability, since it provides higher resolutions compared to publicly available wind datasets and assimilates all available *in situ* data in the region. The atmospheric product was developed at KAUST by the Earth Modeling and prediction group, using the Advanced Research–Weather Research and Forecasting atmospheric model [39]. The atmospheric model was designed with two-way interactive nested domains of 30 and 10 km horizontal resolutions and 35 vertical levels. The inner domain (10 km) covers the Red Sea and adjacent regions. The initial and boundary conditions were obtained from the NCEP Final Analysis (FNL) product. Observations from the NCEP Atmospheric Data Project [40] were assimilated every 6 hours using a consecutive integration approach [41]. The consecutive integration approach uses the forecast as the background in the next assimilation cycle. This provides improved initial conditions for the next integration period, in which the model runs in a free forecast mode. Further details on the experimental design and methodology as well as on the performance of the analysis product are provided in Viswanadhapalli *et al*. [38]. In this study the surface variables are extensively validated with all available observations at different time scales, while regional climatic characteristics are discussed and validated with FNL and different satellite products. Analysis of mean seasonal and monthly winds show that the model accurately reproduces the impact of southwest Indian monsoon flow during summer months. Recent successful applications of this product on Red Sea waves [37,42] and on far-field dispersion of concentrate discharges along the Saudi coast of the Red Sea [43] further support the relevance of this dataset for studying regional climatic characteristics.

### 2.2 Data Analysis

One difficulty associated with the visualization of Chl-a in the Red Sea is the order-of-magnitude variation in annual concentration between the oligotrophic North and the mesotrophic South. This impedes a proper appreciation of the difference in seasonal dynamics between these two areas as well as the spatial propagation of Chl-a. Thus, to compare the seasonal variability of Chl-a in different regions of the Red Sea, we computed the ratio between monthly climatologies and the annual climatology over the 12 years of data available. This allowed us to identify regions where higher Chl-a concentrations occur during a given month. Furthermore, because the statistical distribution of Chl-a data points tends to be skewed towards higher values (Chl-a data is generally log-normally distributed [23]) and outliers can influence the mean considerably, climatologies were computed using the median.

**Fig 3:**
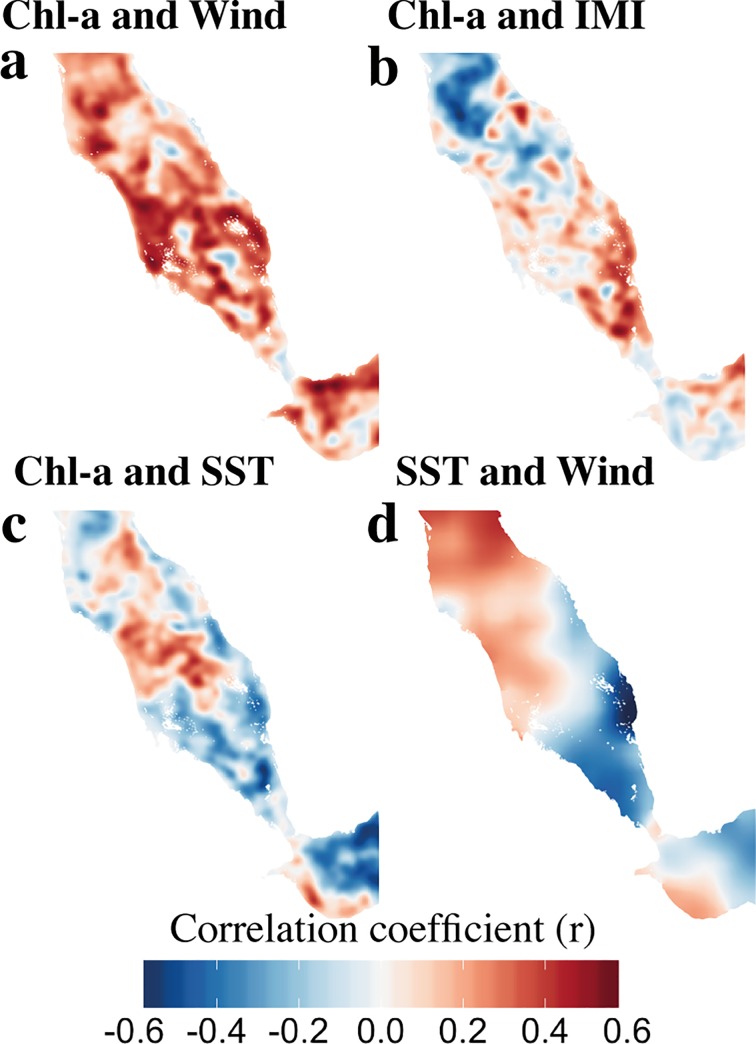
Spatial correlations between Chl-a, SST, wind regime, and IMI in the southern Red Sea during summer. ***a)*** Chl-a and wind regime. ***b)*** Chl-a and IMI. ***c)*** Chl-a and SST. ***d)*** SST and wind regime. The wind regime is defined as the averaged wind speed in the southern Red Sea (below 17°N) and the western Gulf of Aden (west of 46°E). The critical correlation value for a p<0.05 significance level with 11 degrees of freedom is *r* = 0.55.

We produced annual averages of summer (May-August) Chl-a, SST, wind regime and IMI for the southern Red Sea from 2000 to 2012 and used them to compute correlation maps. Chl-a–SST (Fig 3A) correlations were computed on a pixel-per-pixel basis; to obtain a high-resolution map, SST data were projected on the Chl-a grid using the bilinear interpolation method. Correlations shown in Fig 3B–3D were calculated between individual pixels of Chl-a and, respectively, SST individual pixels, the IMI time series and the wind regime. The wind regime was defined as the average of the wind speed in the southern Red Sea (defined as the area below 17°N) and the western Gulf of Aden (west of longitude 46°E, Fig 1A) and represents the strength of the Arabian summer monsoon.

To explore the relative importance of environmental variables, we constructed linear models that predict the 8-day averaged log-concentrations of Chl-a in the southern Red Sea (blue-box area average, Fig 1A) during summer (May to August). The 8-day averaged IMI, wind speed and SST data were used as predictors in the models. SST and wind speed were averaged in the southern Red Sea and the Gulf of Aden (blue- and green-box area averages, respectively, Fig 1A). The regional wind speed and IMI (which accounts for the general pattern of the monsoon winds) were used, since the monsoon winds are the main drivers of upwelling in the Gulf of Aden, which in turn generates the intrusion of nutrient rich water in the southern Red Sea. SST was included in the model, as colder waters in oligotrophic tropical seas are generally indicative of increased nutrient concentrations. Temporal lags of zero, one and two 8-day periods were considered between the predictors and Chl-a concentrations. The performance of the models was evaluated using a cross-validation approach [44]. One year of data was omitted before fitting the model, and was used to compute the corresponding root-mean-square error (RMSE). The averaged RMSE was used to compare the linear model to a climatological prediction. To perform the variable selection and the linear model fitting, we used the lasso regression, a regularized variation of the multivariate linear regression [44]. The lasso regression is a method of variable selection and regularization that forces coefficients which are below a specified threshold to be zero. The objective is to obtain a model that is both simpler to interpret and has better prediction accuracy compared to the classical linear regression.

In the few cases of data gaps (missing pixels) in the Chl-a time-series datasets, the missing values were replaced by the climatological means (based on the 13-year climatology). For the correlation maps, the missing values in the pixel time series were discarded before the correlation computation.

## 3. Results and Discussion

### 3.1 Chlorophyll distribution in the southern Red Sea

**Fig 4:**
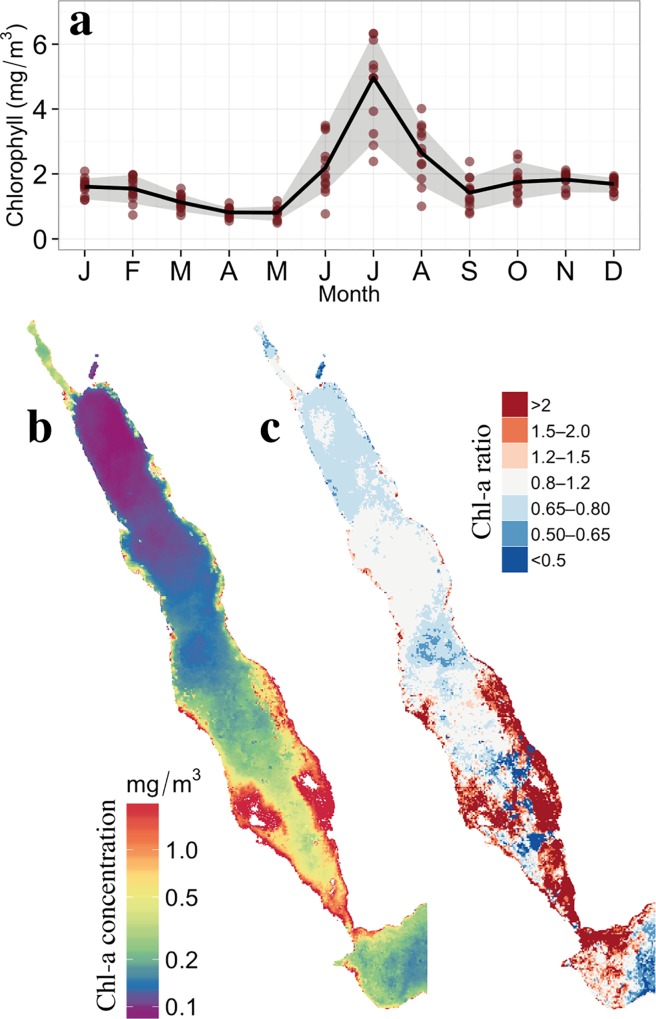
Spatiotemporal distribution of remotely-sensed OC-CCI Chlorophyll in the Southern Red Sea (2000–2012). ***a)*** Monthly climatology of Chl-a averaged in the southern Red Sea (blue-box area average, Fig 1A). The red circular datapoints represent the individual months (2000–2012), while the grey shadow depicts the 90% confidence intervals of the climatology. ***b)*** Spatial distribution of Chl-a during summer; calculated based on monthly climatologies (May to August). ***c)*** Ratio of July to annual Chl-a observations, highlighting the higher concentrations of Chl-a in the southern Red Sea during July; computed from monthly composites between 2000 and 2012.

The recent development and availability of the OC-CCI data product has enabled us to provide a complete description of the southern Red Sea Chl-a concentrations (Fig 1B and 1C) and to reveal the presence of unanticipated intense summer phytoplankton blooms in the southern Red Sea. These blooms exhibit distinct phenological characteristics: they initiate in May, peak in July, and cease in September (Fig 4A). The seasonal climatology also shows that Chl-a concentrations are approximately 250% higher during summer in comparison to winter (*i*.*e*. ~5 mg / m^3^ in July against ~2 mg/m^3^ in January). Fig 4B displays the average concentration of Chl-a during the summer period (May-August, 2000–2012). The summer concentrations are markedly higher in the southern Red Sea, particularly around the Al-Lith, Farasan and Dahlac regions (Fig 4B). However, since the north-south gradient of Chl-a concentration is a well known feature of the Red Sea biology [4,6], we computed the ratio between Chl-a in July and annual climatologies to specifically highlight the summer-blooming regions (Fig 4C, see section 2.2 of methods for details). Overall, the southern Red Sea experiences substantially higher Chl-a concentrations during July compared to the rest of the year. These high summer concentrations occupy a large part of the southern basin, and are particularly pronounced around the eastern shore and Dahlac islands (Fig 4B and 4C).

The 8-day ratio maps computed between 2003 and 2011 (S1 File) show that the summer bloom generally initiates at the end of May/beginning of June in the coastal areas around Al-Lith and the Farasan archipelago. The bloom rapidly expands to the rest of the southern Red Sea, rarely extending beyond 19°N. The distribution of Chl-a exhibits considerable temporal variability during the summer blooming period: Chl-a concentrations tend to reach a maximum in July and terminate in August but the termination date can vary between July and September.

Although Raitsos *et al*. [6] did observe traces of elevated summer Chl-a using MODIS data, its full extent was not anticipated due to the sparseness of satellite Chl-a measurements at that time. A brief temporal analysis of OC-CCI data by Racault *et al*. [11] revealed that many reef-bound areas of the southern Red Sea experience intense summer phytoplankton blooms that are even stronger than the winter ones. Considering the oligotrophic nature of the Red Sea, our analysis of the Chl-a spatiotemporal distribution suggests the existence of a local physical mechanism, capable of supplying sufficient amounts of nutrients to initiate and maintain the blooms. Thus, to understand the southern Red Sea phytoplankton dynamics, it is necessary to investigate the physical processes that facilitate nutrient availability in this region.

### 3.2 Input of nutrient-rich water in the southern Red Sea

#### 3.2.1: Intrusion of nutrient-rich water masses from the Gulf of Aden

The Red Sea is an oligotrophic and highly stratified large marine ecosystem, which mainly relies on its connections with the open ocean for nutrient supply. The only significant water-mass exchange occurs between the Red Sea and the Indian Ocean through the narrow strait of Bab-el-Mandeb. The exchange during winter is that of a typical inverse estuarine circulation; there is an inflow of fresher (less saline) surface water from the Gulf of Aden and a sub-surface outflow of warm, more saline water (Fig 2A and 2B) [8,18,27,45]. During winter, the prevailing winds are orographically constrained to blow northwards over the southern Red Sea, facilitating the intrusion of nutrient-rich surface water from the Gulf of Aden [8,18,27,45].

During summer, the monsoon winds reverse their direction (southwards) in the southern part of the Red Sea, driving an outflow of Red Sea surface water [27,46]. Outside the Red Sea, in the western Gulf of Aden, a corresponding reversing of the prevailing winds (from westward to eastward) is at the origin of an intense upwelling [27,46–48]. Both phenomena (the Red Sea surface outflow and the upwelling in the Gulf) induce the influx of an intermediate water mass from the Gulf of Aden into the Red Sea, known as the Gulf of Aden Intermediate water (GAIW) [27]. In the Gulf of Aden, this water mass is characterized by a low-salinity nutrient-rich layer between 120 m and 420 m depth [49]. Following the wind-induced upwelling in the Gulf of Aden, the core of the GAIW shallows by at least 50 m, facilitating its intrusion into the Red Sea [27,47,49]. The GAIW is nutrient–rich, in contrast to the nutrient-depleted ambient Red Sea waters [50]. As the GAIW enters the Red Sea, it is sandwiched between outflows of surface and deep Red Sea water. Based on direct observations, Sofianos *et al*. [51] estimated that the GAIW intrudes at a rate of 0.3 Sv during the peak of the three-layer exchange at the strait, thus making a significant contribution to the water mass distribution in the area. The three-layer circulation pattern lasts from June to September (during the summer monsoon) [28]. The presence of this phenomenon, during the stratified nutrient-depleted season, could thus provide an important source of nutrients.

Simulations of circulation models indicate that the main part of the GAIW intrusion can reach up to ~16°N in July-August (Fig 2C and 2D). *In situ* salinity and nutrient datasets also show a distinct stream of fresher, nutrient-rich water extending up to 19°N (Fig 2E and 2F). At ~75 m depth, the nutrient concentrations (NO_3_+NO_2_) in the GAIW reached values higher than 10 *μ*mol/l, while salinity was in the range of 37–38 psu (compared to 39 psu for surface water).

**Fig 5:**
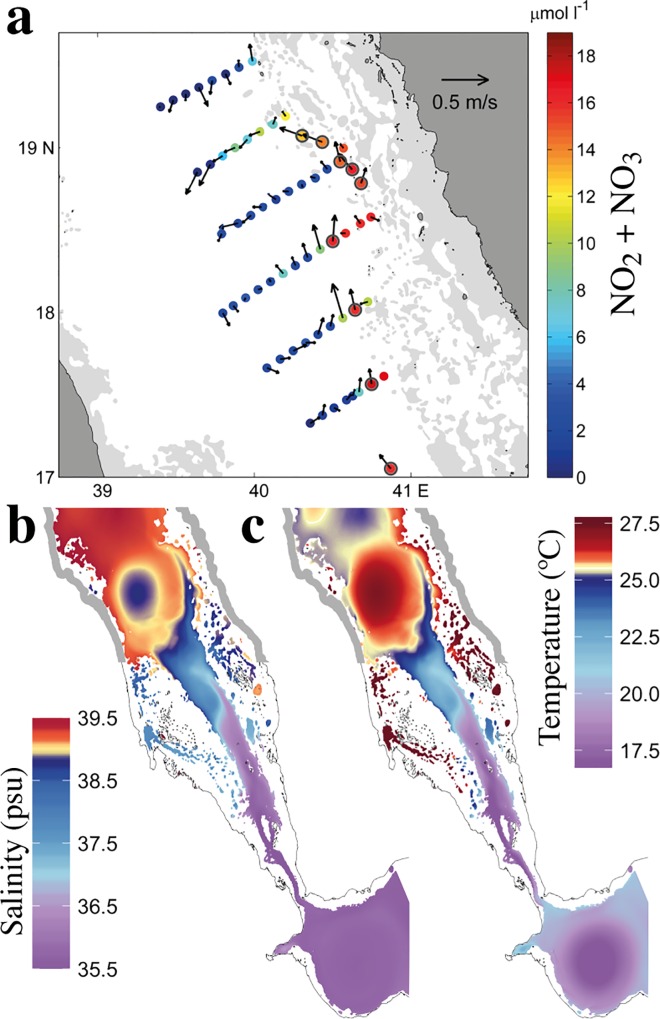
*In situ* nutrient an velocity observations and model outputs of temperature and salinity ***a)*** *In situ* measurements of nutrient concentrations and mean velocity vectors at ~66m depth during September 2011; reprinted and adapted with permission from [18]. ***b)*** *and* ***c)*** September climatological salinity and temperature at 65m depth, calculated from the MITgcm circulation model.

Based on *in situ* measurements of nutrients and velocity fields, acquired at ~66 m depth in September 2011 (Fig 5A, updated from [18]), the nutrient–rich GAIW appears to flow northward, along the Al-Lith banks on the eastern shore (between 17°N and 18°N) [18,27]. To examine this circulation spatially, we display model simulations of salinity and temperature at 65 m depth for the same month (Fig 5B and 5C). Evidently, a layer of fresher and cooler water intrudes into the Red Sea (via the Bab-el-Mandeb strait), and moves northwards towards the shallow areas around the Dahlac and Farasan islands. Above 17°N, the flow is deviated by an anticyclonic eddy and ends around 19°N. Between 17°N and 19°N, the simulated temperature and salinity data (Fig 5B and 5C) and the *in situ* measurements of nutrients (Fig 5A) show a remarkable agreement on the pathway of the intrusion; the GAIW travels northward along the shallow areas of the eastern shore and intrudes in the shallow coral reef areas, delivering nutrients to the local ecosystems. This northward propagation along the eastern side (intrusion path) is clearly evident in the track of higher Chl-a concentrations (Fig 4C).

#### 3.2.2: Interactions and mixing of GAIW with ambient water masses at the Southern Red Sea

**Fig 6:**
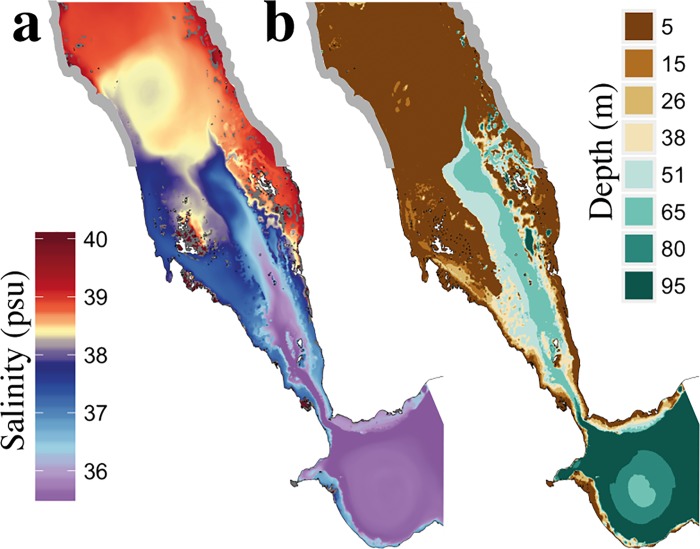
Southern Red Sea simulated minimum salinity between 0m and 100m depth. ***a)*** September climatology of the lowest salinity values observed between 0m and 100m depth—calculated from the MITgcm circulation model, and ***b)*** the depth at which these minimum salinity values occur.

To further elucidate the pathways of the GAIW in the Red Sea, we display the simulated minimum salinity for the layers between 0 m and 100 m depth (Fig 6A). The depth at which this minimum salinity occurs is displayed in Fig 6B. The lowest salinity values, observed at depths between 50 m and 65 m, are representative of the core of the GAIW entering the basin. The model clearly depicts the mixing of fresher waters into the shallow coral reef areas around the Farasan islands. Churchill *et al*. [18] suggested that complex interactions occur between the GAIW stream and coral reefs on the eastern side of the Red Sea. The model simulations reveal that the intrusion of the GAIW in the shallow areas of the Dahlac and Farasan islands takes place between 15°N and 17°N (Fig 6B). The observed higher salinity values around the Farasan islands (compared to the main intrusion path) may be attributed to mixing between the GAIW and the ambient water masses, intensified by the shallow and complex bathymetry of this region.

**Fig 7:**
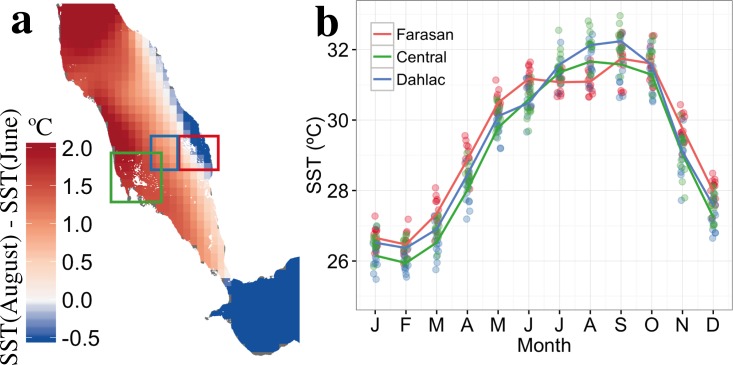
Summer SST climatology in the southern Red Sea, displaying evidences of cooling along the GAIW intrusion pathway. ***a)*** Average SST difference between June and August (2000 to 2012), depicting a cooling of surface waters on the eastern shore of the southern Red Sea. ***b)*** SST monthly climatologies (solid lines) and individual monthly datapoints (solid circles), from 2000 to 2012 for the following regions (displayed in panel a): the rectangles represent areas in Farasan archipelago (red), central southern Red Sea (blue), and Dahlac archipelago (green).

The seasonal climatology of remotely-sensed SST provides further evidence of mixing occurring between the GAIW and the surface layers (Fig 7A). A noticeable decrease in SST occurs around the Farasan islands between June and August, whereas temperatures generally increase in the rest of the southern Red Sea. Overall, the regions experiencing a decrease (or a slight increase) in SST between June and August (Fig 7B) correspond to the regions of high Chl-a observed off the eastern shore (Fig 4B and 4C). They also coincide with the pathway followed by the GAIW intrusion along the eastern shore up to ~19°N, further supporting the notion that mixing occurs between the GAIW and shallow waters near the Farasan islands and Al-Lith banks. It is thus evident that areas characterized by regionally colder SST are associated with higher Chl-a concentrations, implying the transfer of nutrients to the upper layers due to mixing processes.

In this section, we used simulated temperature and salinity data, to provide a complete description of the pathway of the GAIW intrusion and its mixing within the coral reef systems surrounding the Dahlac and Farasan islands. Remotely-sensed SST data confirm that the GAIW mixes with surface water along the eastern shore, whilst spatial maps of Chl-a reveal the occurrence of phytoplankton blooms along the pathway of intrusion. It is therefore highly likely that the GAIW intrusion plays a key role in facilitating regional phytoplankton blooms.

### 3.3 Interannual variability of phytoplankton blooms in relation to the physical environment

#### 3.3.1 Correlations between Chl-a and environmental parameters

In an attempt to clarify the relationship between the summer phytoplankton blooms in the southern Red Sea and the GAIW intrusion, we further examine the Chl-a interannual variability in relation to selected environmental variables. Specifically we examine the spatial correlations between summer (May–August) averages of Chl-a, SST, wind speed and the IMI (see methods in section 2.2). SST variability allows us to explore the relationship between the presence of colder waters (which are potentially richer in nutrients) and higher Chl-a concentrations. Analysis of the relationship between Chl-a and wind intensity (and Chl-a and IMI) allows us to infer the influence of wind-driven mechanisms.

The positive correlations between the average regional wind speed and the summer Chl-a (evident across most of the southern Red Sea, Fig 3A) highlight the persistent relationship between the wind regime and summer phytoplankton blooms. The interaction between the wind regime and Chl-a is coherent with the proposed mechanism that links wind intensity, the GAIW intrusion and phytoplankton activity; intense surface winds tend to increase the strength of the intrusion of nutrient-rich GAIW. High positive correlations between Chl-a and the IMI are also observed during summer, along the path of the GAIW intrusion from the Bab-el-Mandeb strait to the Farasan islands (Fig 3B). These results further highlight the relationship between the local environment and the Arabian monsoon dynamics, through the teleconnection of the local wind regime.

Significant negative correlations are also observed between averaged summer SST and Chl-a (Fig 3C, r = -0.57, p < 0.05, based on blue-box area average [Fig 1A]). This is particularly evident in areas where high Chl-a concentrations occur, such as the Farasan/Dahlac archipelagos and the Al-Lith bank (below 17°N), and where mixing occurs along the eastern shore, as revealed from the climatology analysis of remotely-sensed SST data (Fig 7A). Thus, it is evident that the intrusion of GAIW in the shallow areas and the transfer of nutrients to the upper layers through mixing are typical features of the GAIW pathway.

Strong negative correlations are also evident between SST and wind speed in areas of the southern Red Sea that experience enhanced Chl-a concentrations (Fig 3D). The correlations are strongest along the eastern shore, close to the Farasan islands, where a large decrease in SST is observed during summer (Fig 7B). The spatial pattern of the correlation between SST and wind intensity coincides with the pathway of GAIW into the Red Sea and the high Chl-a concentrations, and thus links the two processes: the wind-regulated intensity of the nutrient-rich GAIW intrusion and the local processes that may supply the available nutrients to the upper layers.

Collectively, the results of the correlation analysis support the hypothesis that the intrusion of nutrient-rich GAIW in the southern Red Sea contributes to the formation of large summer phytoplankton blooms; stronger monsoon winds enhance the upwelling in the western part of the Gulf of Aden, which subsequently enhances the GAIW influx into the southern Red Sea [46,51]. The GAIW intrudes into the shallow coral reef areas where it interacts with ambient water masses (as hypothesised by Churchill *et al*. [18]) and reaches the surface layers. As evidenced by the corresponding reduction of SST (Fig 7), this interaction supplies the surface waters with nutrients, enhancing phytoplankton growth. These interactions are more pronounced in the reef complexes along the eastern shore (i.e. along the pathway of the intrusion along the eastern shore of the southern Red Sea [18,27]).

#### 3.3.2 Predictive model of weekly Chl-a concentrations

To assess the relative importance of different environmental variables (wind, IMI, and SST) on Chl-a concentrations, linear models were fitted to predict the weekly averaged Chl-a in the southern Red Sea. The lasso regression (see methodology for further information) was used to select the most explanatory variables. The parameters that showed significant correlations with Chl-a were used as predictors in the model: SST, wind speed and the IMI. Wind speed was averaged in the southern Red Sea and in the Gulf of Aden (blue- and green-box area averages respectively, Fig 1A), while SST was averaged in the southern Red Sea only. The variables were taken with lags of zero, one or two 8-day periods.

**Fig 8:**
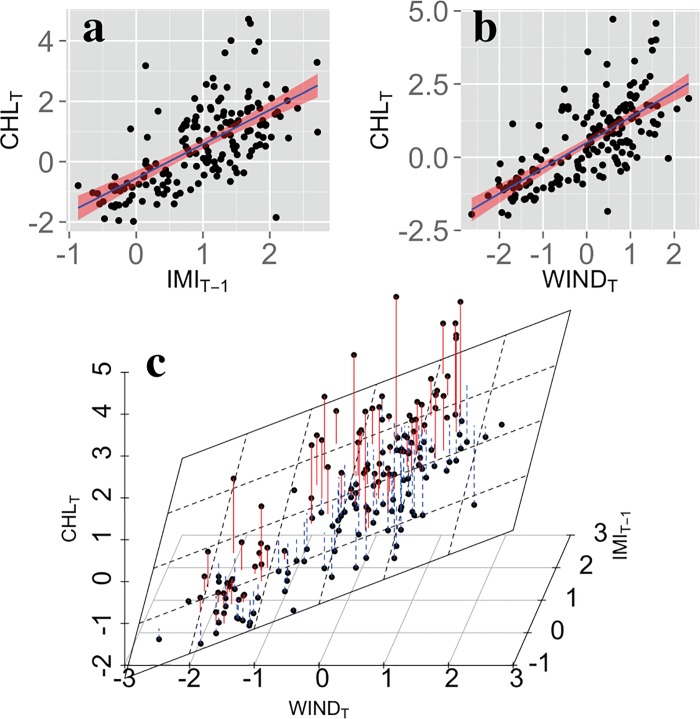
Statistical models of summer Chl-a averages over the southern Red Sea. ***a)*** Linear regression model of weekly Chl-a using IMI with a one-week lag as predictor. ***b)*** Linear regression model of weekly Chl-a using the wind speed averaged in the Gulf of Aden (west of 46°E) as predictor. ***c)*** Multivariate linear regression surface (white plane) of weekly Chl-a with the final predictors of wind speed and IMI. Positive and negative model errors on the data are represented with red and blue lines respectively. The solid black circles represent the datapoints.

Among the variables used in the regression analysis, the final model selected the averaged wind speed over the Gulf of Aden and the IMI (with a lag of 1 week) to be the most important, explaining 52% of Chl-a variability (Fig 8A and 8B). The resulting 2-dimensional model is displayed in Fig 8C. It is evident that Chl-a concentrations increase in response to stronger monsoonal winds (as indicated by IMI) and regional winds (over the Gulf of Aden), both of which drive the regional upwelling in the Gulf of Aden. The model selection of the wind over the Gulf of Aden instead of the wind over the southern Red Sea suggests that the effect of monsoonal winds on the upwelling dominates over local wind effects. The elimination of the averaged wind speed in the southern Red Sea does not exclude a possible impact of local winds on the nutrient supply to the upper layers, through vertical mixing or upwelling in the region. However, local mixing/upwelling in the absence of GAIW, would not produce the same effects, since ambient water masses at similar depths in the Red Sea have significantly lower nutrient concentrations (Fig 2). The 8-day period lag between IMI and Chl-a reflects the spatial distance between the Red Sea and the locations where the index is calculated (between the Indian Ocean and northern India). The elimination of SST by the model can be attributed to the fact that the variability is mainly driven by the monsoon reversal and intense regional winds.

Finally, although the relationships Chl-a–IMI (Fig 8A), and Chl-a–wind speed (Fig 8B) appear slightly exponential, we did not find statistical evidence of nonlinear relationships between the predictors and Chl-a (after fitting a generalized additive model [44]). To show the existence of such nonlinearities, additional data or a more sophisticated model is required.

## 4. Conclusion

Previous attempts to assess satellite-derived summer phytoplankton biomass in the southern Red Sea have been constrained by the persistent presence of clouds, which severely limits the available number of observations [6]. Using a recently developed multi-sensor dataset (OC-CCI), with significantly improved data coverage, we provided the first complete description of the spatiotemporal distribution of previously reported intense phytoplankton blooms in the southern Red Sea [11]. These blooms, which are more intense than the winter blooms, generally begin in May, peak in July and cease between August and September. They first appear in the shallow areas close to Al-Lith and the Farasan islands and then rapidly expand to the rest of the southern Red Sea (south of 19°N).

The southern Red Sea phytoplankton blooms are regulated by the circulation patterns at the Bab-el-Mandeb strait. The reversal of the Asian summer monsoon facilitates the intrusion into the southern Red Sea of an intermediate layer of colder, fresher, nutrient-rich water from the Gulf of Aden [18,27]. Using *in situ* and simulated datasets, we show that this intermediate water generally travels northward, mostly along the eastern shore of the basin, and intrudes into the shallow areas around the Dahlac and Farasan islands. This nutrient-rich water mass then mixes with the ambient waters of the coral reef systems surrounding the Dahlac and Farasan islands, as shown by remotely sensed SST and Chl-a datasets. Our statistical analysis is consistent with the hypothesis that the combination of the aforementioned physical mechanisms regulates the supply of the euphotic zone with nutrients, leading to intense summer phytoplankton blooms in the southern Red Sea. This is the first attempt to describe and explain these summer blooms (as seen from space), and further multidisciplinary approaches (using *in situ*, modeled and remotely-sensed observations) are needed to identify nutrient pathways and study their impact on the marine algae of the Red Sea.

## Supporting Information

S1 File Ratio of weekly to annual Chl-a observations between 2000 and 2012, showing the succession of winter and summer blooms and their spatio-temporal variability. (MPEG)

S2 File Monthly climatological profiles of salinity from the MITgcm circulation model averaged over the transect indicated indicated in Fig 1A and used to obtain Fig 2B and 2D. (MAT)

S3 File Monthly climatological profiles of temperature from the MITgcm circulation model averaged over the transect indicated indicated in Fig 1A and used to obtain Fig 2A and 2C. (MAT)

S4 File 8-day averaged data Chl-a, SST, wind speed, MEI, IMI (with different lags) used to fit the lasso regression model to forecast Chl-a over the southern Red Sea (Fig 8). (CSV)

S5 File Monthly (2000–2012) wind speed data from the Weather Research and Forecasting atmospheric model, used to obtain Fig 3A and 3D. (ZIP)
